# The outcome of oncoplastic techniques in defect reconstruction after resection of central breast tumors

**DOI:** 10.1186/s12957-015-0688-1

**Published:** 2015-09-26

**Authors:** Omar Farouk, Essam Attia, Sameh Roshdy, Ashraf Khater, Ahmad Senbe, Adel Fathi, Emad-Eldeen Hamed, Mahmoud Mesbah, Fayez Shehatto, Aiman El-Saed, Adel Denewer

**Affiliations:** Surgical Oncology Department, Oncology Center, Faculty of Medicine, Mansoura University, Mansoura, Egypt; Community Medicine Department, Faculty of Medicine, Mansoura University, Mansoura, Egypt

**Keywords:** Central breast cancer, Oncoplastic breast surgery, Conservative breast surgery, Breast reconstruction, SSM, Grisotti technique

## Abstract

**Background:**

Traditionally, conservative breast surgery was contraindicated in centrally located breast tumors, with total mastectomy as the treatment of choice. However, restorations of the central defects by the oncoplastic volume displacement or replacement techniques have been shown to be effective. The aim of the current study was to assess the surgical outcome of oncoplastic techniques after central breast tumors resection.

**Methods:**

Thirty patients with central breast cancer, including two patients with Paget disease, treated at the Oncology Center of Mansoura University (Egypt) between June 2011 and December 2014 were included in this study. The oncoplastic techniques performed were Grisotti advancement rotational flap in eight (26.7 %) patients, classic skin-sparing mastectomy (SSM) with latissimus dorsi pedicled flap in 20 (66.7 %) patients, and skin-reducing mastectomy (SRM) with latissimus dorsi pedicled flap using wise pattern inverted T incision in two (6.7 %) patients. The choice of the oncoplastic techniques depends on the achievement of free safety margins, the breast volume, and its ptotic degree.

**Results:**

The median age was 40.5 years (range; 23–55). There were no major complications that require repeating the oncoplastic techniques. Recorded complications included wound dehiscence (4/30, 13.3 %) donor site seroma (4/30, 13.3 %), and surgical site infection (1/30, 3.3 %). The 6-month subjective patient satisfaction was excellent in 21 (70 %) patients, good in 6 (20 %) patients, and fair in 3 (10 %) patients. There was no episode of local recurrence or systemic metastasis after an average follow-up duration of 24 months (range; 6–42).

**Conclusions:**

Restoring the central defect after resection of the central breast tumors can be safely achieved using oncoplastic procedures including the Grisotti technique or the design of SSM or SRM with immediate breast reconstruction. In our patients, these procedures yield a satisfactory esthetic outcome with lower morbidity.

## Background

Centrally located breast tumors represent 5–20 % of all breast cancer cases [1]. Traditionally, conservative breast surgery was contraindicated in these tumors with total mastectomy as the treatment of choice. This may be attributed to the fear of local control failure and risk of tumor multicentricity [2, 3].

Additionally, the conventional conservative treatment or central quadrantectomies, which includes excision of the nipple-areola complex (NAC) and the correspondent underlying cylinder of parenchyma down to the pectoralis fascia, may result in local glandular defects and poor esthetic outcome including obvious distortion of breast contour and scar contracture in most cases. However, restoration of the central defect by the oncoplastic volume displacement or replacement techniques has been shown to be effective.

The choice of the oncoplastic techniques depends on the achievement of free safety margins, the breast volume, and its ptotic degree. For example, oncoplastic volume displacement such as Grisotti technique is suitable in ptotic breasts while reduction vertical mammoplasty is suitable for large and huge breasts. Additionally, volume replacement techniques including total breast reconstruction is suitable in cases of small breast and/or non-ptotic breasts or when the safety margins could not be achieved in conservative surgery.

The objective of the current study was to assess the short-term surgical and esthetic outcomes of oncoplastic techniques after centrally located breast tumor resection.

## Methods

### Design

A prospective study was carried out at the Oncology Center of Mansoura University (Egypt) during the period between June 2011 and December 2014. All females with central breast tumors during the study period who were candidates for restoration of the central defect by either oncoplastic volume displacement or replacement techniques were asked to join the study. Required ethical approval was obtained from local ethical committee and written informed consents were obtained from all patients before enrollment (Medical Research Ethics Committee of Faculty of Medicine in Mansoura University).

Thirty patients with centrally located breast cancer, including two patients with Paget disease of the nipple, were enrolled in this study. Four patients with stage III received neoadjuvant chemotherapy according to MDT decision. They showed partial response after the sixth cycle and had SSM with latissimus dorsi (LD) myocautaneous flap.

The exclusion criteria included extensive skin involvement outside the area of NAC, multicentricity, inflammatory carcinoma, distant metastasis, and patients who refused breast reconstruction. Demographics, tumor characteristics, and oncoplastic techniques among the study patients are summarized in Table 1.

**Table 1:** Demographics, tumor characteristics, and oncoplastic techniques among the study patients (*N* = 30)

	Number^a^	Percentage
Patients age (years)		
Median	40.5	
Range	23–55	
Tumor pathology		
Paget disease of the nipple	2	6.7
Ductal carcinoma in situ (DCIS)	1	3.3
Invasive ductal carcinoma	24	80.0
Invasive lobular carcinoma	2	6.7
Medullary carcinoma	1	3.3
Tumor stage		
Stage 0 (non invasive)	3	10.0
Stage I	4	13.3
Stage II	19	63.3
Stage III	4	13.3
Oncoplastic techniques		
Grisotti advancement rotational flap	8	26.7
SSM with LD myocautaneous flap	20	66.7
SRM with LD myocautaneous flap	2	6.7

*SSM* skin-sparing mastectomy, *SRM* skin reducing mastectomy, *LD* latissimus dorsi

^a^Unless mentioned otherwise

### The oncoplastic surgical plane

1. If the breast was large and ptotic and the tumor resected has free safety margins, Grisotti advancement rotational flap was applied (eight patients).
2. If the breast was small and/or non-ptotic breasts or when the safety margins could not be achieved during resection, classic skin sparing mastectomy (SSM) was applied with immediate total breast volume replacement (reconstruction) using LD myocautaneous flap (20 patients).
3. If the breast was large and ptotic and the safety margins could not be achieved during resection, type IV SSM (wise pattern inverted T incision or skin-reducing mastectomy (SRM)) was applied with immediate total breast volume replacement (reconstruction) using LD myocautaneous flap (two patients).

### Oncoplastic surgical techniques

**Fig. 1:**
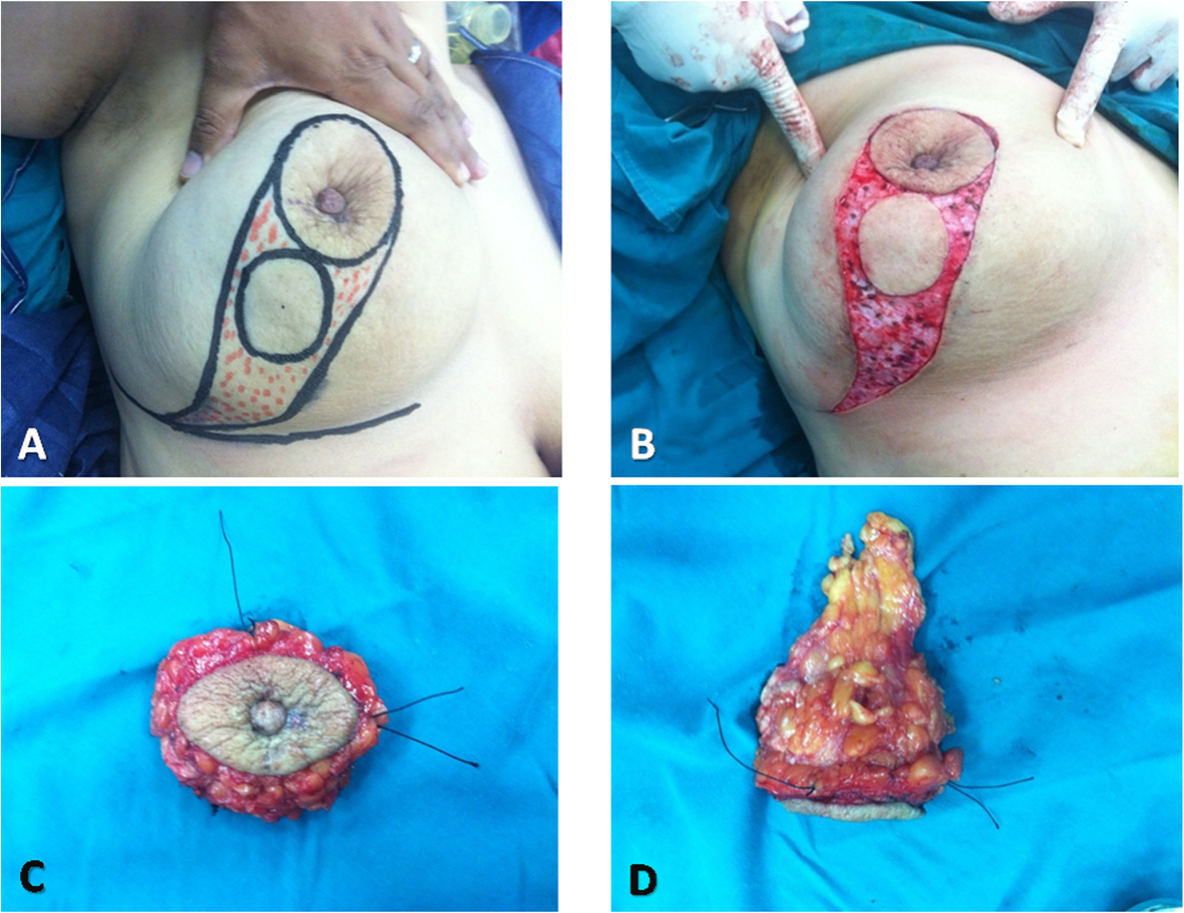
Grisotti technique: **a** Marking of the NAC outline with another smaller circle being just below the NAC within a comma-shaped appearance. **b** Complete de-epithelialization of the flap (except the new areola). **c** Top view of the central quadrantectomy including NAC and tumor with marking the specimen peripheries using threads for intra-operative frozen section analysis. **d** Lateral view of the same central quadrantectomy showing a column of tissue from the subcutaneous layer down to the pectoral fascia
  Central quadrantectomy including NAC and tumor with a column of tissue from the subcutaneous layer down to the pectoral fascia was done (Figs. 1c, d and 2a) with marking the specimen peripheries for intra-operative frozen section analysis (Fig. 1c, d).

1. Grisotti technique
  The operation started with marking of the NAC outline, another smaller circle being just below the NAC, and also the inframammary sulcus; then, the medial and lateral borders of the flap were drawn extending from the medial and lateral margins of the areolar down to the inframammary fold and converging distally to give a comma-shaped appearance (Fig. 1a). Then complete de-epithelialization of the flap (except the new areola) was done (Fig. 1b).

**Fig. 2:**
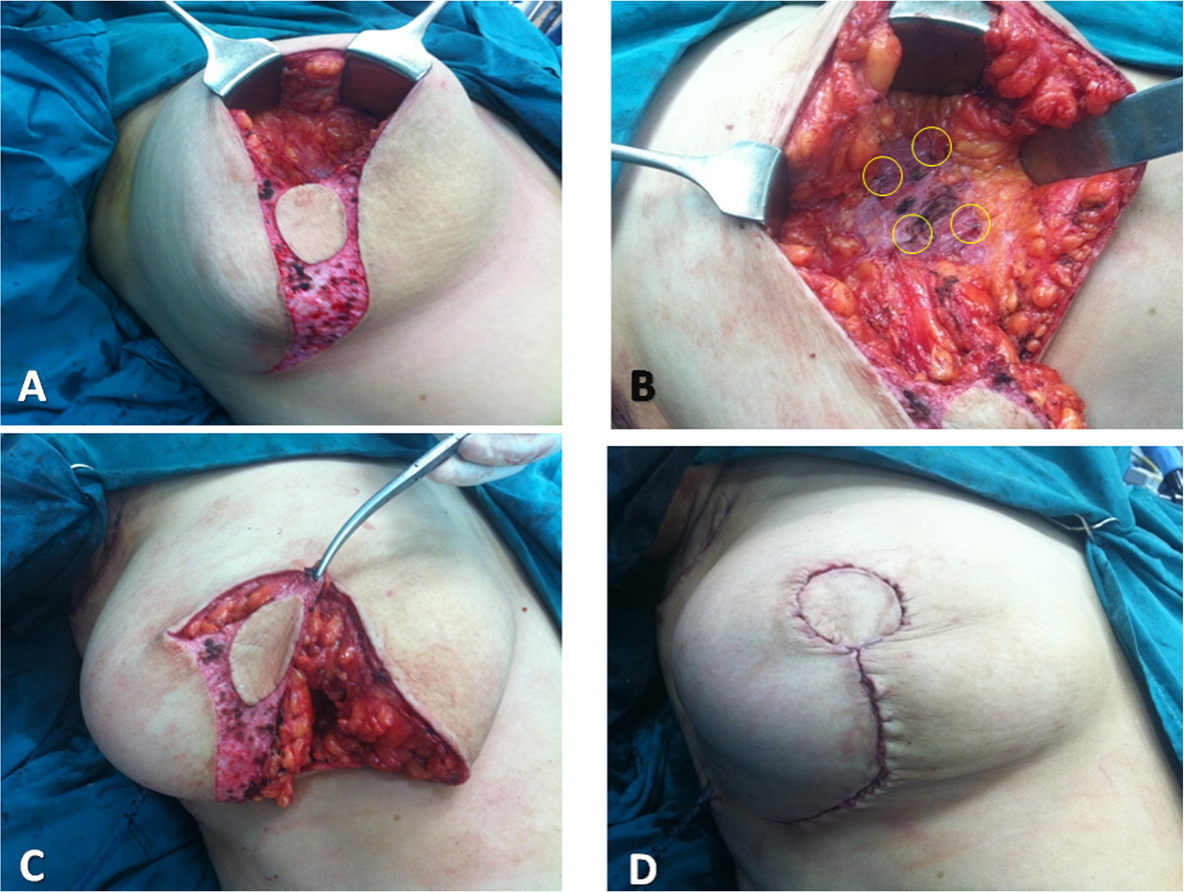
Grisotti technique (continued): **a** The tumor bed after central quadrantectomy. **b** Four titanium clips were placed along the margins of the tumor bed to facilitate subsequent adjuvant radiotherapy. **c** The medial and inferior margins of the flap were then incised down to the pectoral fascia with wide mobilization of the flap from the pectoral fascia. **d** The flap was advanced and rotated to fill the defect with complete suture of the wounds
  Four titanium clips were placed along the margins of the tumor bed to facilitate subsequent adjuvant radiotherapy (Fig. 2b). The medial and inferior margins of the flap were then incised down to the pectoral fascia with wide mobilization of the flap from the pectoral fascia; then, the flap was advanced and rotated to fill the defect (Fig. 2c) with complete suture of the wounds (Fig. 2d). Another separate incision in the axillary fold was done for axillary LN dissection. The same procedure was done for Paget disease of the nipple (Fig. 3).

**Fig. 3:**
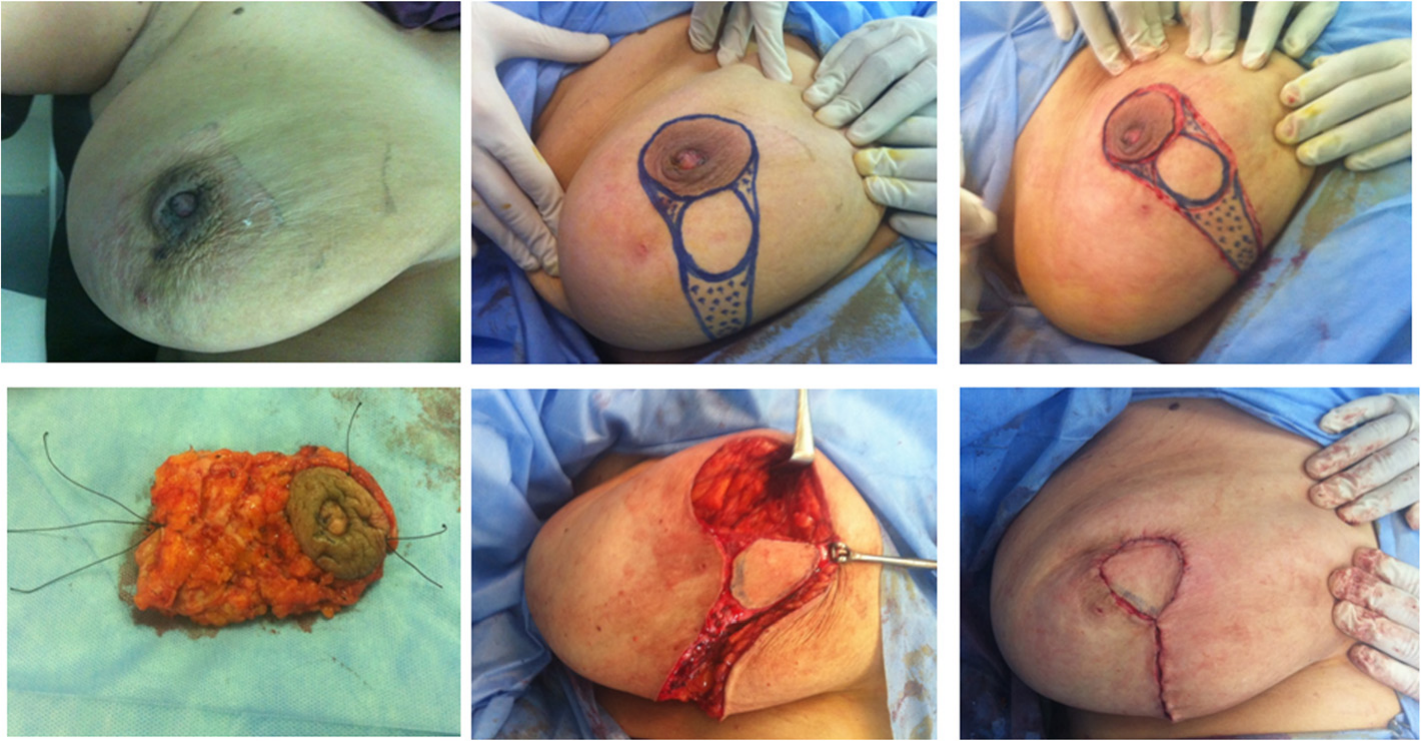
Another patient with Paget disease of the nipple and Grisotti technique
2. Skin sparing mastectomy (SSM) and immediate breast reconstruction (IBR)
  The operation started with periareolar incision including NAC (Fig. 4a); through it, the dissection of whole breast parenchyma and axillary lymph node dissection could be achieved (Fig. 4b–d).

**Fig. 4:**
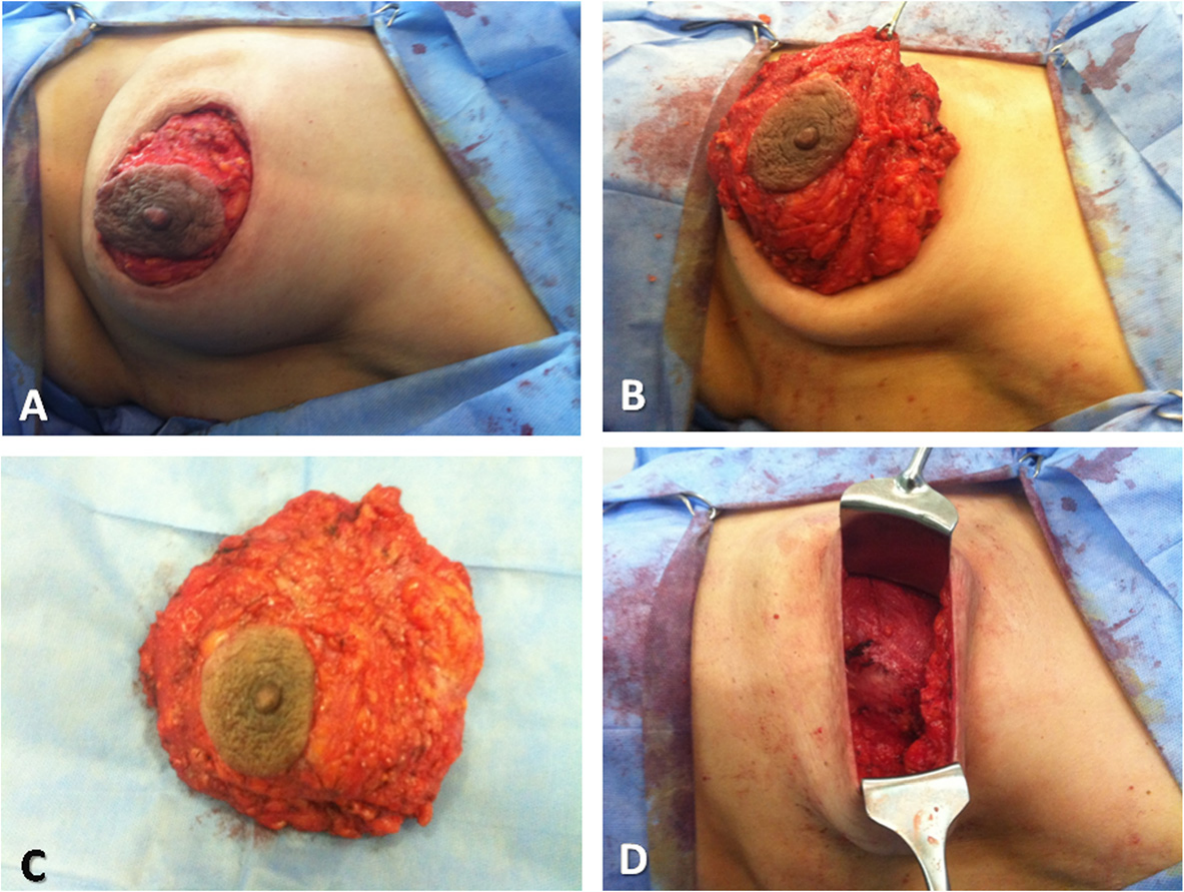
Skin sparing mastectomy (SSM) and immediate breast reconstruction (IBR): **a** Periareolar incision including NAC. **b** Dissection of whole breast parenchyma. **c** The whole breast specimen. **d** The breast skin envelop after complete breast parenchymal resection
  Then, the patient was turned to her side position and a transverse incision including a skin paddle at the back was designed (Fig. 5a), and then, the dissection of the whole latissimus dorsi was performed and the incision was closed (Fig. 5b); then, the patient was turned again to supine position, the latissimus dorsi myocautaneous flap was transposed through the axillary tunnel to the breast envelope (Fig. 5c), the breast mound was reconstructed, and the periareoal incision was closed with the skin paddle (Fig. 5d).

**Fig. 5:**
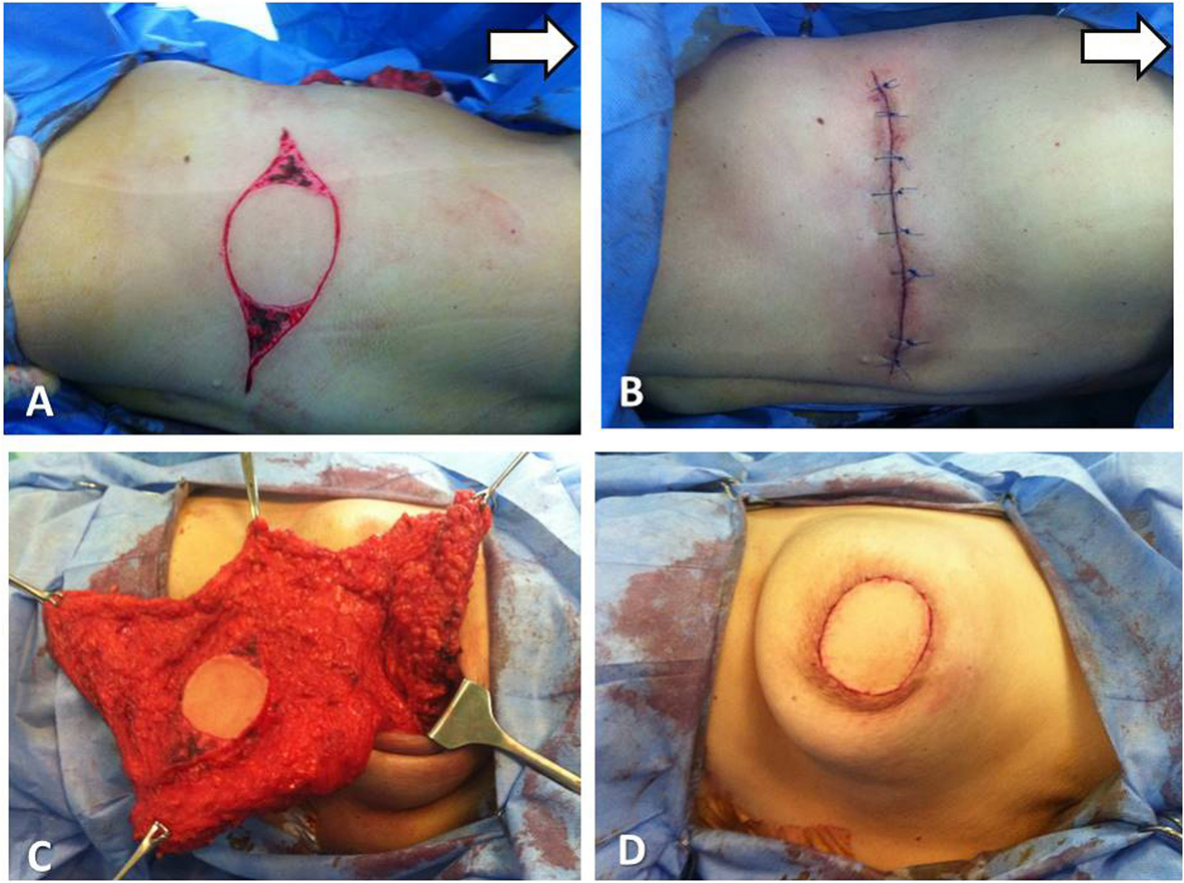
Skin sparing mastectomy (SSM) and immediate breast reconstruction (IBR) (continued): the *arrow* refers to the head position. **a** The transverse incision including a skin paddle at the back. **b** Closure of the back incision after complete dissection of latissimus dorsi. **c** Transposition of the latissimus dorsi myocautaneous flap through the axillary tunnel to the breast envelope. **d** Reconstruction of the breast mound closure of the periareolar incision with the skin paddle

### Study outcome

Postoperative complications were reported, and all patients were evaluated 6 months after surgical operation, in order to evaluate the esthetic outcomes which were judged by both surgeon and patient satisfactions. The subjective patient satisfaction about her reconstructed breast was expressed as excellent (five points), good (four points), fair (three points), poor (two points), and very poor (one point).

All patients were referred to clinical oncology department in order to receive adjuvant therapy; 21 patients received adjuvant chemotherapy and 13 received radiotherapy.

## Results

### Patient and tumor characteristics

The median age was 40.5 years (range; 23 to 55). The most common pathologic diagnosis was invasive ductal carcinoma (24/30, 80 %), followed by Paget disease of the nipple and invasive lobular carcinoma (two patients each). The most common tumor stage was stage II (19/30, 63 %), followed by stages I and III (four patients each).

### Oncoplastic techniques

The oncoplastic techniques performed were Grisotti advancement rotational flap in 8 (26.7 %) patients, SSM with latissimus dorsi pedicled flap in 20 (66.7 %) patients, and SRM with latissimus dorsi pedicled flap in 2 (6.7 %) patients. Patients who were in need of contralateral surgery in order to achieve a standard symmetry refused to do any contralateral surgery. Also patients with SSM refused to do tattooing of areola and nipple reconstruction.

### Postoperative complications

Recorded complications included wound dehiscence (4/30, 13.3 %) and surgical site infection (1/30, 3.3 %). Wound dehiscence was treated by secondary suturing, and surgical site infection was treated conservatively. Donor site seroma was seen in four patients among those that had latissimus dorsi flap reconstruction and was managed conservatively by frequent aspiration. However, There were no major complications, either in the skin envelop or loss of the reconstructed flaps, that require repeating the oncoplastic techniques. The short-term surgical complications as well as esthetic and oncologic outcomes among the study patients are shown in Table 2.

**Table 2:** Short-term surgical complications as well as esthetic and oncologic outcomes among the study patients (*N* = 30)

	Number^a^	Percentage
Short-term surgical complications		
Wound dehiscence	4	13.3
Donor site seroma	4	13.3
Surgical site infection	1	3.3
Subjective patient satisfaction with esthetic outcome		
Excellent	21	70.0
Good	6	20.0
Fair	3	10.0
Follow-up duration (months)		
Mean	24	
Range	6–42	
Oncologic outcome by end of follow-up duration		
Recurrence	0	0.0
Metastasis	0	0.0

^a^Unless mentioned otherwise

### Esthetic and oncologic results

**Fig. 6:**
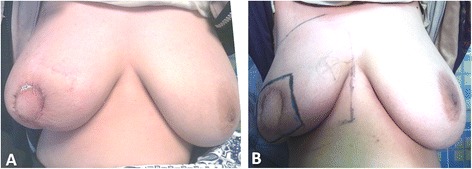
Postoperative views of Grisotti technique: **a** after 3 weeks and **b** after 6 months with good esthetic outcome. The patient was prepared for radiotherapy

**Fig. 7:**
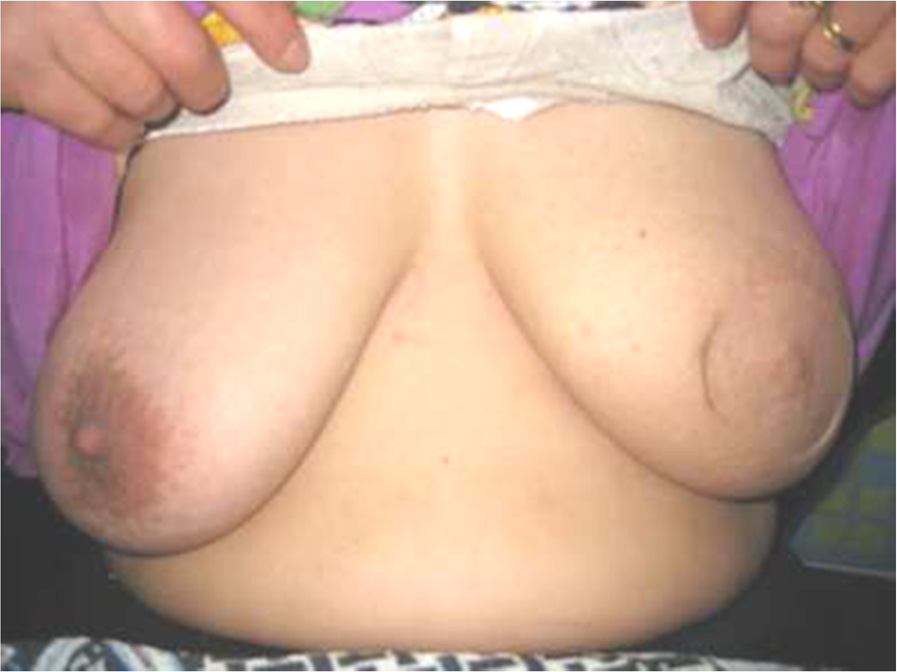
Postoperative views of SSM with latissimus dorsi myocautaneous flap after 12 months with excellent esthetic outcome. The patient refused nipple reconstruction, areola tattooing, and contralateral surgery for symmetry

The 6-month subjective patient satisfaction was excellent in 21 (70 %) patients, good in 6 (20 %) patients (Figs. 6 and 7), and fair in 3 (10 %) patients. There was no episode of local recurrence or systemic metastasis after an average follow-up duration of 24 months (range; 6 to 42).

## Discussion

Audretch has defined the oncoplastic techniques as all approaches of plastic and reconstructive surgery aimed at achieving tumor resections with satisfactory margins in conservative treatment, preserving the natural shape and appearance of the breast, attempting to minimize potential deformities, and to obtain the best possible cosmetic results [4].

In other detailed words, Schrenk has described oncoplastic techniques as the resection of the tumor (either partial or total mastectomy) and immediate reconstruction of the defect using plastic surgical techniques (local parenchymal/muscle flaps or free flaps). It includes many different techniques like excision of the cancer with adequate free margins to achieve loco-regional control, immediate remodeling of the defect to improve the cosmetic result, contralateral breast symmetrization and reconstruction of the NAC when needed, and immediate and late reconstruction after mastectomy [5].

For central breast cancer, the resection of the NAC is always necessary and cosmetic results are mostly poor if standard breast conservative therapy is performed. Thus, most surgeons perform a mastectomy in patients with central breast cancer [6]. This may be based on personal oncologic concerns and the often poor cosmesis resulting from resection of the NAC and subareolar glandular tissue [7].

We believe that conservative management of central breast cancers using resection of the central breast cancers without breast remodeling using an elliptical excision is a simple and safe solution; however, loss of breast projection is an important disadvantage that will be more pronounced the more volume that is resected [8].

Therefore, central resection with breast remodeling should be reserved for patients with discrepancy between the volume of the resection and the size of the breast; however, there are wide varieties of remodeling options in oncoplastic techniques. The pattern of selection depends primarily on the breast’s anatomical characteristics like size and ptotic level. For example, a single-skin pattern is indicated in medium breasts with moderate ptosis since breast remodeling will provide the best results in these circumstances. For a voluminous breast and/or one with moderate/severe ptosis, a double-skin onco-reductive mammoplasty is recommended since this procedure can significantly reduce the breast volume and sagging which will optimize postoperative irradiation [8].

We are reporting a generally mild postoperative complications and a very acceptable esthetic outcome among a group of Egyptian women diagnosed with central breast tumors and were candidates for restoration the central defect by either oncoplastic volume displacement or replacement techniques.

We did not carry out any implant reconstruction. This may be attributed to many factors: (1) Most of Egyptian ladies prefer to have autologous breast reconstruction rather than implant reconstruction because they believe that the feeling of the breast becomes more natural with autologous reconstruction rather than implants. (2) The cost of autologous breast reconstruction is cheaper than implant reconstruction because the Egyptian health insurance do not cover the cost of implants. (3) The quality of radiotherapy machines and techniques are relatively limited because of limited resources, so Egyptian surgical oncologists prefer to do autologous breast reconstruction rather than implant reconstruction in order to decrease the expected radiotherapy related complications which are associated with implants.

The esthetic outcome in the current study was very acceptable, as indicated by the high percentage of those rated the 6-month outcome as excellent (70 %) or good (20 %). This may be attributed to our accumulated learning curve of practice in SSM and NSM with IBR using LD flaps, which started since 2004 in Oncology Center—Mansoura University [9, 10]. Similarly, previous studies showed a very good patient acceptance. For example, Wagner et al. examined the patient satisfaction of 33 patients after breast-conserving surgery (BCS) in the form of central quadrantectomy with complete removal of the NAC which reveled excellent in 80 % and good in 20 % with no poor result [7].

In the current study, no episodes of local recurrence or systemic metastasis were reported. This may be attributed to relatively small number of patients that is coming from the fact of relative small percentage of central breast tumors, in addition to the short time of follow-up (mean 24). However, the oncologic outcome of BCS was shown to be comparable to the classic mastectomy in cases of central breast tumors. For example, after a median follow-up of 42 month of 69 patients who underwent either BCS or mastectomy, there was no difference between both groups with respect to local breast or axillary recurrence, systemic metastases, or disease-related death [7].

## Conclusions

Despite the challenges in restoring the central defect after resection of the central breast tumors, using the oncoplastic procedures either the Grisotti technique for large ptotic breasts or the design of SSM or SRM with immediate breast reconstruction when conservative breast surgery is contraindicated, are considered acceptable alternatives to mastectomy and yield a satisfactory esthetic outcome with lower morbidity. However, further long-term study is needed to assess the long-term outcome of these surgical procedures in terms of survival.

### Consent

Written informed consent was obtained from the patient for the publication of this report and any accompanying images.

## References

[1] Grisotti A, Casella D, Calabrese C, Fitzal F, Schrenk P (2010). Immediate reconstruction of central quadrantectomy defects with a rotation flap—the Grisotti technique. Oncoplastic breast surgery; a guide to clinical practice.

[2] Simmons RM, Brennan M, Christos P, King V, Osborne M (2002). Analysis of nipple/areolar involvement with mastectomy: can the areola be preserved?. Ann Surg Oncol.

[3] Vlajcic Z, Zic R, Stanec S, Lambasa S, Petrovecki M, Stanec Z (2005). Nipple-areola complex preservation: predictive factors of neoplastic nipple-areola complex invasion. Ann Plast Surg.

[4] Audretsch W, Rezai M, Kolotas C (1994). Oncoplastic surgery: “target” volume reduction, (BCT mastopexy) lumpectomy reconstruction (BCT reconstruction) and flap supported operability in breast cancer. Proceedings of the second European congress on senology.

[5] Schrenk P, Fitzal F, Schrenk P (2010). Oncoplastic breast surgery. Oncoplastic breast surgery; a guide to clinical practice.

[6] Fitzal F, Nehrer G, Hoch D, Riedl O, Gutharc S, Deutinger M (2007). An oncoplastic procedure for central and medio-cranial breast cancer. EJSO.

[7] Wagner E, Schrenk P, Huemer GM, Sir A, Schreiner M, Wayand W (2007). Central quadrantectomy with resection of the nipple-areola complex compared with mastectomy in patients with retroareolar breast cancer. Breast J.

[8] Nebril BA (2009). Oncoplastic techniques in the management of central breast cancer. CIR ESP.

[9] Denewer A, Farouk O (2007). Can nipple-sparing mastectomy and immediate breast reconstruction with modified extended latissimus dorsi muscular flap improve the cosmetic and functional outcome among patients with breast carcinoma?. World J Surg.

[10] Denewer A, Setit A, Hussein O, Farouk O (2008). Skin-sparing mastectomy with immediate breast reconstruction by a new modification of extended latissimus dorsi myocutaneous flap. World J Surg.

